# Improved tumour delivery of iron oxide nanoparticles for magnetic hyperthermia therapy of melanoma *via* ultrasound guidance and ^111^In SPECT quantification†

**DOI:** 10.1039/d4nr00240g

**Published:** 2024-07-17

**Authors:** P. Stephen Patrick, Daniel J. Stuckey, Huachen Zhu, Tammy L. Kalber, Haadi Iftikhar, Paul Southern, Joseph C. Bear, Mark F. Lythgoe, Simon R. Hattersley, Quentin A. Pankhurst

**Affiliations:** a Centre for Advanced Biomedical Imaging (CABI), Department of Medicine, University College London London WC1E 6DD UK peter.patrick@ucl.ac.uk; b Healthcare Biomagnetics Laboratory, University College London 21 Albemarle Street London W1S 4BS UK; c Resonant Circuits Limited 21 Albemarle Street London W1S 4BS UK; d School of Life Science, Pharmacy & Chemistry, Kingston University Penrhyn Road Kingston upon Thames KT1 2EE UK

## Abstract

Magnetic field hyperthermia relies on the intra-tumoural delivery of magnetic nanoparticles by interstitial injection, followed by their heating on exposure to a remotely-applied alternating magnetic field (AMF). This offers a potential sole or adjuvant route to treating drug-resistant tumours for which no alternatives are currently available. However, two challenges in nanoparticle delivery currently hinder the effective clinical translation of this technology: obtaining enough magnetic material within the tumour to enable sufficient heating; and doing this accurately to limit or avoid damage to surrounding healthy tissue. A further complication is the lack of established methods to non-invasively quantify nanoparticle biodistribution, which is necessary to evaluate the performance of improved delivery strategies. Here we employ ^111^In radiolabelling and single-photon emission computed tomography (SPECT) to non-invasively quantify distribution of a clinical grade iron-oxide-based nanoparticle in a mouse model of melanoma. We show that compared to manual injection, ultrasound guided delivery together with syringe-pump-controlled infusion improves both the nanoparticle concentration within the tumour, and the accuracy of delivery – reducing off-target peri-tumoural delivery. Following AMF heating, injected melanomas shrank significantly compared to non-injected controls, validating therapeutic efficacy. Systemic off-target delivery was quantified and extrapolated to predict off-target energy absorbance within safe limits for the main sites of background accumulation. With many nanoparticle-based therapies currently in development for cancer, this image-guided delivery strategy has wide potential impact beyond the field of magnetic hyperthermia. Future use in representative patient cohorts would also be enabled by the high clinical availability of both SPECT and ultrasound imaging.

## Abstract 2

We increased the accuracy of tumour nanoparticle delivery using real-time ultrasound imaging and slow syringe-pump controlled infusion. This minimises off-target side effects for better and safer melanoma treatment using magnetic hyperthermia.

## Graphical abstract

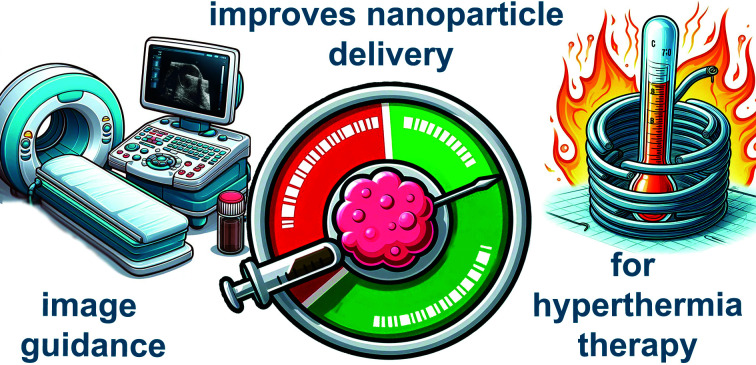

## Introduction

Hyperthermia therapies exploit the increased sensitivity of tumours to super-physiological temperatures, using sustained heating between 41 and 45 °C or above to increase apoptosis in cancer cells compared to healthy tissues.^1,2^ This increased heat-sensitivity of tumours has been explained by a range of differences in their biochemistry (*e.g.* increased reactive oxygen species), cell signalling (*e.g.* dysregulated homeostasis pathways), and anatomy (*e.g.* disorganized vasculature reducing the heat sink effect).^3–8^ Yet, sustained heating of normal tissue must also be avoided to prevent unwanted side-effects, with necrosis typically affecting healthy human tissue when sustained above 50 °C for over 5 minutes.^2,9^

Magnetic field hyperthermia (MFH) is a particularly attractive approach to hyperthermia therapy, compared to less localised, or whole-body hyperthermia approaches. MFH relies on the ability of alternating magnetic fields (AMFs) to selectively heat magnetic nanoparticles – which may be delivered into the tumour tissue by either direct (*e.g.* interstitial) or indirect (*e.g.* systemic) administration routes – while leaving the surrounding or adjacent healthy tissue volumes unaffected.^10,11^ By allowing faster and higher local tumour heating and response rates compared to most externally applied heating sources, including radio-frequency and microwave radiation, it reduces the duration of therapy needed, and the potential for damaging side-effects to healthy tissues.^11–13^ However, for nanoparticle-based hyperthermia to work effectively in practice, relatively high concentrations (1 to 50 mg iron per mL tumour volume) must be accurately delivered to the tumour, preferably with an even coverage, and while minimizing off-target delivery to nearby locations. For example, in a recent clinical study of MFH as part of the treatment of pancreatic cancer, the target administration concentration of the magnetic nanoparticles used was *ca.* 25 mg of iron per mL of tumour tissue, which was projected to be sufficient to deliver 0.66 W g_tissue_^−1^ into the tumour when subjected to an AMF of amplitude 4 kA m^−1^ and frequency 330 kHz.^14^

With varying success, several indirect (intravenous) administration strategies have been attempted to address this challenge of accurate delivery, including: magnetic targeting,^15,16^ antibody functionalization,^17^ and using tumour-homing stem cells as carriers.^18^ Various cancer type or scenario-specific direct administration routes have also been tested, including: gel or paste application to resected tumour margins,^19,20^ visually-guided manual intra-tumoural injection,^21^ inhalation for lung tumours,^17^ stereotactic delivery for glioblastoma,^22^ catheterisation of tumour-supplying arteries, and ultrasound and/or endoscopy guidance for interstitial injection into liver, pancreatic,^23^ and prostate tumours.^21^

Direct comparison of particle delivery achieved using different delivery methods can, however, be challenging, as the endogenous contrast of iron oxide particles is not easily quantifiable, with non-invasive imaging methods such as X-ray CT being generally too insensitive in the clinic (poor contrast at concentrations < 10 mg_Fe_ mL_tissue_^−1^), or, conversely, oversensitive, as with MRI (contrast saturation and artefacts at concentrations > 1 mg_Fe_ mL_tissue_^−1^).^15,24^ On the other hand, Magnetic Particle Imaging (MPI) is specifically designed to quantify superparamagnetic iron oxide particles such as those used for MFH, and has both suitable dynamic range and high enough resolution for this purpose.^25^ Despite these advantages and the growing preclinical availability of MPI, work is still ongoing to scale-up hardware before it can be used clinically.^26,27^ The current state of the field therefore leaves some guesswork involved in deciding which delivery technique is best to use in clinical trials, and a need for quantitative technologies to fill this gap. Aside from evaluating delivery methods, post-injection quantification of particle delivery and dispersion is also important to plan AMF heating schedules and parameters, including power output and heating duration, which depends on the amount of iron in the tumour of each patient (or experimental animal in the case of preclinical studies). These parameters must be controlled to achieve sufficient heating across the tumour to ensure a therapeutic effect, and to avoid damage to adjacent or surrounding normal tissue.

As a potential solution for this challenge of non-invasive *in vivo* quantification, we recently investigated a rapid and simple labelling method for attaching radionuclides to the surface of magnetic nanoparticles.^28^ This was found to neither affect their bulk magnetic nor chemical properties, while giving comparable biodistribution to unmodified stock particles. This offers the potential for accurate and non-invasive measurement of the delivered nanoparticle dose across the body using nuclear imaging modalities such as SPECT (single photon emission computed tomography, radiolabelling with indium-111) and PET (positron emission tomography, radiolabelling with zirconium-89), both of which have a higher dynamic signal range and orders of magnitude greater sensitivity than either MRI or CT. This approach also has good prospects for clinical translatability due to the wide availability of nuclear imaging hardware, and the simplicity of the radiolabelling procedure.

We apply this technique here to quantify the tumour delivery efficacy *via* different interstitial administration routes, including manual needle placement and injection, ultrasound guidance with manually controlled injection rate, and ultrasound guidance with syringe pump-controlled injection rate. For translational relevance, throughout this study we use the clinical grade magnetic nanoparticle formulation RCL-01,^29^ and a preclinical model of melanoma.

The RCL-01 magnetic heating agent consists of *ca.* 140 nm diameter multicore magnetic nanoparticles containing *ca.* 10 nm diameter magnetic iron oxide cores (predominantly maghemite, γ-Fe_2_O_3_) encapsulated in a dextran matrix. These nanoparticles were developed by Resonant Circuits Limited, London, UK. They have a substantial magnetic heating capacity, characterised by an intrinsic loss power (ILP) metric^30^ of order 5 nH m^2^ kg_Fe_^−1^, and have previously been shown to reduce tumour volumes in an orthotopic preclinical model of pancreatic cancer following hyperthermia treatment.^29^ A safety and feasibility focused clinical study (the “NoCanTher” study^31^) using these same RCL-01 agent is currently under way in Barcelona, exploring their use alongside standard-of-care chemotherapy for the treatment of non-resectable locally-advanced pancreatic cancer.

Future application of the RCL-01 agent (and similar products in development) in other cancer types is envisaged where current treatment options are inadequate. One example of this is melanoma, where, despite recent breakthroughs in immunotherapy (including immune checkpoint inhibitors) that have greatly improved treatment options, disease resistance still typically emerges in the majority of advanced stage (IV) patients.^32,33^ This has led to specific calls for intra-tumoural combination therapies that directly kill cancer cells, releasing tumour-specific neo-antigens to boost the anti-tumour T-cell response.^33,34^ Although beyond the scope of the current work (where experiments were performed using an immune-suppressed animal model), it is important to note that such a mechanism can induce abscopal effects, priming the immune response against metastases distant from the treatment site. This effect has been previously achieved with hyperthermia for a range of tumour types, enhancing the anti-cancer potency of both immunotherapies and the native immune system.^2,35–39^ Though clinical studies combining hyperthermia with immunotherapy for melanoma are yet to emerge, it has been suggested as one of the most promising potential indications for this synergistic approach.^40^

Here we established a mouse model of melanoma and compared a range of nanoparticle delivery options for their effective dose delivered (initial retention), and accuracy (target *versus* off-target delivery). In particular we compared the tumoural *versus* peri-tumoural distribution of the injected nanoparticles, and also the total nanoparticle mass retained in the tumour, relative to the tumour size and administered dose. Ultrasound guided delivery combined with slow controlled injection using a syringe-pump was found to give the highest accuracy and total dose to tumours compared to manual dosing, or ultrasound guidance alone. Application of AMFs to the successfully injected tumours resulted in a significant decrease in tumour volume as measured by CT, compared to control non-injected AMF-exposed tumours in the same animals, demonstrating the utility of this approach. Ultrasound-guided, syringe-pump-controlled delivery, coupled with radiolabelling-enabled post-administration *in vivo* quantification, provides a clinically translatable real-time method for accurate patient-specific dosing. This may be useful to enhance the response rate of other magnetic heating agents, or even cell-based therapies in cancer or regenerative medicine.

## Results

### RCL-01 nanoparticles retain their magnetic and heating properties after indium-111 (^111^In) or zirconium-89 (^89^Zr) radio-labelling

Multicore magnetic nanoparticles (RCL-01, Resonant Circuits Limited) comprising *ca.* 10 nm maghemite cores dispersed in a *ca.* 140 nm diameter dextran matrix were labelled with ^111^In and ^89^Zr using the heat-induced radiolabelling method (a.k.a. “radio-mineralisation”), as described previously.^28,41^ This gave radiochemical yields of 96.3% (*n* = 3, SD = 2.5%) and 98.1% (*n* = 3, SD = 1.7%) respectively as measured by thin layer chromatography (TLC), consistent with our previous findings with similar nanoparticles.^28^ Demonstrating the role of heating in the labelling process, otherwise identical control samples were incubated at room temperature (instead of 90 °C) with ^111^In or ^89^Zr. This showed no appreciable labelling, with comparable retention values to radiometal stock solutions (Table S1†).

**Fig. 1:**
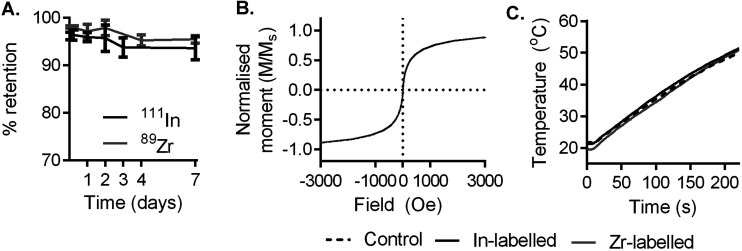
Radiolabelling is stable and does not affect the magnetic or heating properties of RCL-01 nanoparticles. (A) RCL-01 magnetic nanoparticles labelled with ^111^In and ^89^Zr *via* surface radio-mineralisation retain activity during up to 1 week of incubation at 37 °C in human serum. In and Zr-labelled RCL-01 particles (5 mg_Fe_ mL^−1^) behaved comparably to control (unmodified) stock particles in terms of (B) magnetic properties as measured by SQUID magnetometry, and (C) heating induced by a time-varying magnetic field of amplitude 8.5 kA m^−1^ and frequency 990 kHz.

To evaluate the stability of radiolabel attachment in a biologically relevant environment, samples produced as above were incubated in human serum at 37 °C for 1 week (Fig. 1A). During this time, radiolabel attachment was found to be stable, with 93.6% (*n* = 3, SD = 2.4%) and 95.4% (*n* = 3, SD = 0.8%) of ^111^In and ^89^Zr remaining on the particles respectively, as shown by TLC, after 7 days.

We then used SQUID (superconducting quantum interference device) magnetometry to monitor the effect of labelling on the particles’ magnetic properties. RCL-01 labelled with InCl_3_ or ZrCl_4_ (natural abundance isotopes) showed comparable magnetization curves and saturation magnetization to unmodified stock control particles (Fig. 1B and Fig. S1A–C†), showing no effect of the labelling process on their magnetic behaviour. Examination with transmission electron microscopy (TEM) found no discernible changes in chemical behaviour or size distribution as a result of the heating process or labelling with In or Zr (Fig. S2A–F†). This was also consistent with our previous findings with similar nanoparticles.^28^

To evaluate their heating performance post-labelling, 5 mg_Fe_ mL^−1^ solutions of stock, and labelled (In, and Zr) particles were exposed to an AMF (amplitude 8.5 kA m^−1^, frequency 990 kHz, Materials Characterisation MACH system, Resonant Circuits Limited) and the temperature measured over 4 minutes using a thermal probe. Heating rates were comparable across samples, showing that the labelling process had no deleterious impact on the potential therapeutic effect of the magnetic nanoparticles (Fig. 1C).

The *in vivo* labelling stability following the radio-mineralisation method was then confirmed, following subcutaneous injection of ^89^Zr-RCL-01, by monitoring the ^89^Zr PET signal and its correspondence to the CT contrast of the electron dense iron oxide core up to 8 days post injection (Fig. S3 and S4†). Whereas free (unbound) ^89^Zr is known to accumulate in the bone,^42^ no evidence of this was detected up to 8 days post injection, providing a further confirmation of labelling stability. Illustrating the advantages of nuclear imaging over MRI, a hyperthermia relevant dose of RCL-01 (5 mg_Fe_ mL^−1^) was found to show saturation of MR signal hypo-intensity, and surrounding signal artefacts (Fig. S4†), while producing clear PET-CT images. ^111^In was used for all further *in vivo* imaging due to its wider clinical availability, and the higher resolution of preclinical SPECT *versus* PET.

### Melanoma xenograft model

A mouse melanoma xenograft model was generated by implanting human A-375 malignant melanoma cells subcutaneously in the contralateral rear flanks of immunocompromised (SCID) mice. Tumour growth was monitored using caliper measurements and X-ray CT. Tumours were palpable from day *ca.* 13 post-implantation. CT was found to be an accurate measure of tumour volume, with a correlation of *R*^2^ = 0.92 with the wet weight of excised tumours (*n* = 12 tumours). Volumes derived from caliper measurements were found to be less accurate, with an *R*^2^ of 0.65 (*n* = 28 tumours) showing weaker correlation with wet excised weight, consistent with a previously published study on subcutaneous tumour measurement.^43^ CT measurements were therefore used for the remainder of the study.

### Ultrasound guidance provides increased particle delivery and better accuracy

The degree of heating achievable with magnetic field hyperthermia is strongly dependent on the amount of magnetic material delivered to the target tissue. Hence successful treatment relies on an accurate initial injection, with sufficient material delivered, and retained at the time of heating. The duration of heating, and thus the effective dose to the tumour, is also limited by the amount of off-target delivery, which risks damage to surrounding healthy tissue. We therefore assessed three protocols for intratumoural injection to evaluate their accuracy (tumour *versus* peri-tumoural delivery), and efficacy (percentage of the target dose delivered to the tumour). To measure the amount of material in the tumour, and around it, we used ^111^In-labelled RCL-01 magnetic nanoparticles (as described above) and SPECT-CT imaging, to provide whole-body biodistribution data and region of interest quantification.

To determine injection volumes, the tumour volume was measured before injection using CT, with one third of this volume taken as the target injection volume of RCL-01 magnetic heating agent. This ratio has previously been estimated as the optimal dose for interstitially delivered magnetic fluid hyperthermia formulations, taking into account predicted dispersion and retention factors estimated from the known behaviour of established clinical diagnostic iron-oxide based nanoparticles.^44^ This is also consistent with volumes used previously in the clinic, which have ranged from 20 to 40% of the total tumour volume across a range of tumour types including rectal, cervical, and prostate cancers, and glioblastoma.^21,22,45^

**Fig. 2:**
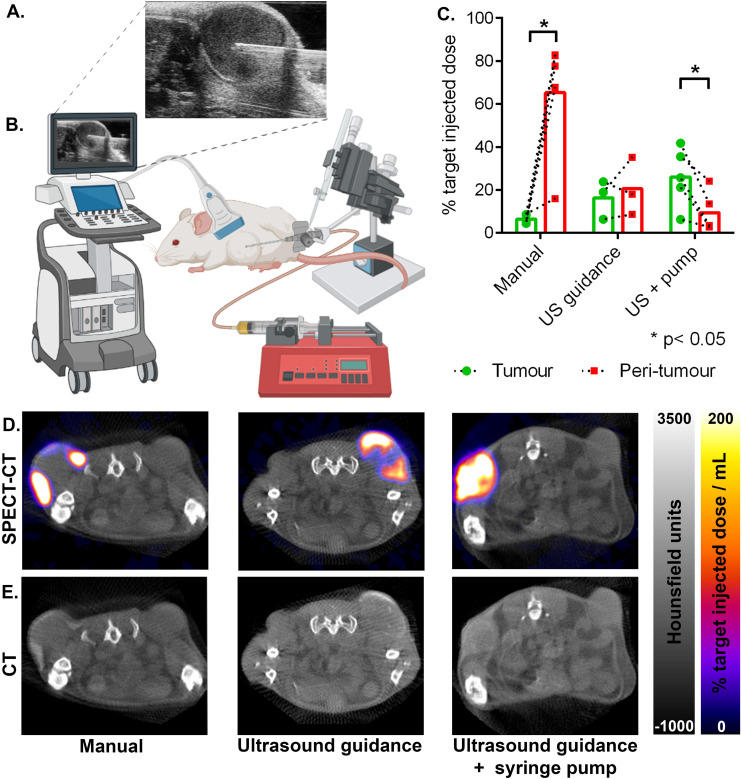
Ultrasound guidance and slow infusion *via* syringe-pump enhances intra-tumoural delivery while minimising peri-tumoural off-target delivery. (A) Ultrasound snapshot taken during real-time guidance of the needle to the centre of the tumour. (B) Schematic of the delivery equipment including ultrasound system, 3-axis micro-manipulator, and syringe pump, catheter and needle. (C) Quantification of the particle delivery to intra-tumoural and peri-tumoural locations using SPECT imaging at 1 hour post injection for 3 different delivery strategies: manual needle placement and injection, ultrasound guided needle placement with manual depression of the syringe, and ultrasound guided needle placement with syringe-pump controlled infusion (as shown in panel B). Points show individual paired intra-tumoural and peri-tumoural replicates (*n* = 3 to 5 animals per condition), bars show means. (D) Representative ^111^In SPECT-CT and (E) CT images taken 1 hour post injection, showing transverse sections through contra-lateral control and target tumours. ^111^In-labelled RCL-01 particle location appears as both SPECT signal and as a hyper-intense CT signal.

The results of the tumour delivery efficacy experiments are summarised in (Fig. 2C–E). Here SPECT signal (Fig. 2D) was quantified with region of interest analysis, using the CT (Fig. 2E) image to provide anatomical context and demarcate tumour from peri-tumour. Manual placement of the needle with manual injection gave the lowest tumour delivery (6% ± 2% SD), with significantly more of the target injected dose being in the peri-tumoural zone (65% ± 28% SD), giving the lowest accuracy (*p* = 0.011, 2-tailed paired *t*-test). Ultrasound guidance (Fig. 2A and B) together with syringe-pump controlled infusion rate gave the highest tumour delivery (26% ± 14% SD), with significantly more nanoparticles in the tumour than in the off-target peri-tumoural region (9% ± 9% SD), and with the highest accuracy (*p* = 0.018, 2-tailed paired *t*-test). Ultrasound guidance without the use of the syringe pump gave an intermediate amount of tumour delivery (16% ± 9% SD), with no significant difference *versus* the peri-tumoural region (21% ± 14% SD), showing an intermediate accuracy (*p* = 0.57, 2-tailed paired *t*-test).

Comparison of the amount of ^111^In-RCL-01 delivered to the tumour showed that both the ultrasound-guidance alone, and the ultrasound guidance together with syringe pump-controlled infusion gave significantly higher nanoparticle concentrations in the tumour than with manual needle placement and injection (Fig. 2C; *p* = 0.047, and *p* = 0.013 respectively, 2-tailed unpaired *t*-tests). In addition to this, they also gave a lower percentage of the target dose in the peri-tumoural region compared to manual injection (*p* = 0.046, and *p* = 0.003 respectively, 2-tailed unpaired *t*-tests), thereby lowering the potential for damage to healthy tissue during hyperthermia therapy (Fig. 2C–E).

As the method with the highest amount of tumour nanoparticle delivery, as well as the highest accuracy (target *vs.* off-target delivery), the use of ultrasound guidance with syringe-pump controlled infusion should result in the least damage to healthy tissue, and the best treatment response. Therefore, this technique was taken forward to investigate the effect of AMF application on tumour heating, and its effect on tumour growth.

### Magnetic hyperthermia reduces tumour volume

**Fig. 3:**
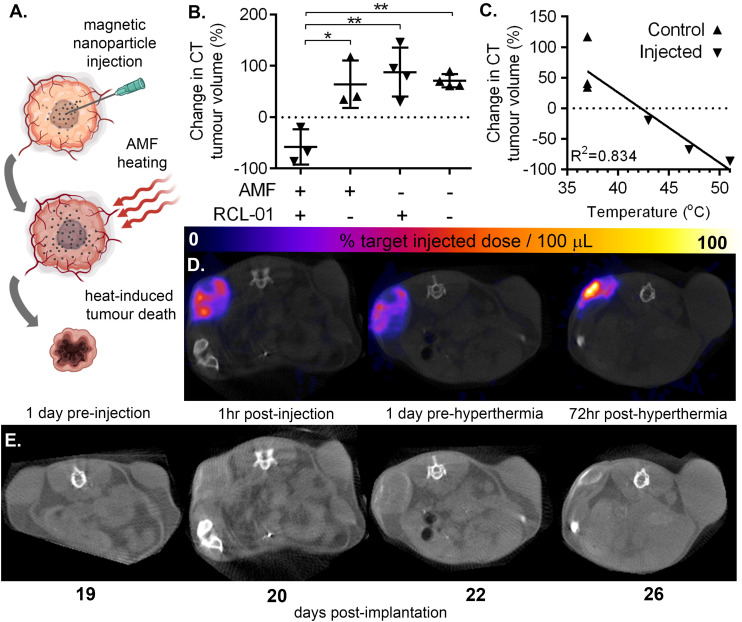
Magnetic particle injection and AMF-heating causes tumour shrinkage in correlation with peak measured temperature. (A) Schematic of magnetic nanoparticle hyperthermia therapy. (B) Tumour volume, measured with CT 1 day before, and 72 hours post hyperthermia therapy showed a significant difference in change of size between RCL-01-injected tumours compared to non-injected contralateral control tumours in the same mice also exposed to AMF (1-tailed paired *t*-test, *p* = 0.042), and *versus* injected (*p* = 0.0034) and non-injected (*p* = 0.00045) tumours not exposed to AMF (unpaired, 1-tailed *t*-tests). (C) Change in tumour size measured between 1 day before treatment and 72 hours post treatment correlated with the peak measured surface temperature of the tumours during the hyperthermia therapy. (D) Transverse SPECT-CT and (E) CT sections from a representative mouse, showing increase in control tumour size (upper right), and decrease in the injected tumour size (upper left), together with the distribution of the injected ^111^In-RCL-01 nanoparticles (positive contrast).

Following intratumoural delivery of RCL-01 with ultrasound guidance and syringe pump-controlled infusion we assessed the heating response, and therapeutic effect of AMF exposure (Fig. 3A) using a magnetic field generator designed especially for animal experiments (Preclinical MACH system, Resonant Circuits Limited). To minimise the risk of damage to healthy tissue we measured tumour temperatures for an initial 10 minutes during AMF at a low amplitude of 4.21 kA m^−1^ and frequency 940 kHz, predicted to be below that needed for therapeutic heating, resulting in no measurable increase in temperature. This was followed by 20 minutes at a higher amplitude of 5.47 kA m^−1^ at 940 kHz, which was predicted to be sufficient for therapeutic heating, and led to measurable increases in surface temperature of nanoparticle injected tumours, but not control (uninjected) contralateral tumours.

The results of these experiments are summarised in Fig. 3 and Fig. S5.† Comparison of tumour volumes as measured by CT 1 day pre hyperthermia and 3 days post hyperthermia treatment showed a significant difference in growth rate between those tumours injected with RCL-01 (Fig. 3B–E; a mean decrease by a factor 0.58 ± 0.34 SD), and those not injected with RCL-01 (a mean increase by a factor 1.64 ± 0.36 SD). Strong negative correlation of tumour growth rate with peak measured surface temperature was also found (*R*^2^ = 0.834), with the two tumours that shrank the most also recording the greatest heating (Fig. 3C). In contrast to the injected tumours, the non-injected (control) tumours showed no heating during exposure to AMF, and grew in size following treatment, consistent with the assumption that the presence of RCL-01 was necessary to achieve heating (Fig. 3B and E). Further to this, we also found a good, but weaker correlation (*R*^2^ = 0.767) between treatment effect over the same time frame (% change in tumour size) and estimated iron content in the tumours (mg_Fe_ mL_tissue_^−1^), based on SPECT quantification of particle distribution (Fig. S5†).

Interestingly we measured wide variation in tumour growth rates before hyperthermia (doubling times between 48 and 243 hours), with better treatment response relative to iron amount seeming to trend with slower growth. Better treatment prediction modelling might therefore be achieved in the future by combining such biological information with the physical metrics of iron concentration and distribution.

The maximal iron concentration measured here was *ca.* 2.6 mg_Fe_ mL_tissue_^−1^, which, given that the RCL-01 magnetic particles have an intrinsic loss power (ILP) of *ca.* 5 nH m^2^ kg_Fe_^−1^, corresponds to a specific absorption rate (SAR) in the tumour tissue of *ca.* 0.37 W g^−1^, at the higher, 5.47 kA m^−1^ AMF setting. To control for the effect of nanoparticle injection alone, tumour size was monitored in the same way following injections of RCL-01 in mice not exposed to AMF (Fig. 3B), showing no significant difference between the growth of contralateral tumours (*p* = 0.304, 1-tailed paired *t*-test). Also as expected, exposure to AMF alone showed no effect on tumour size (*p* = 0.825, unpaired 2-tailed *t*-test).

### *Ex vivo* confirmation of nanoparticle location and treatment effect

**Fig. 4:**
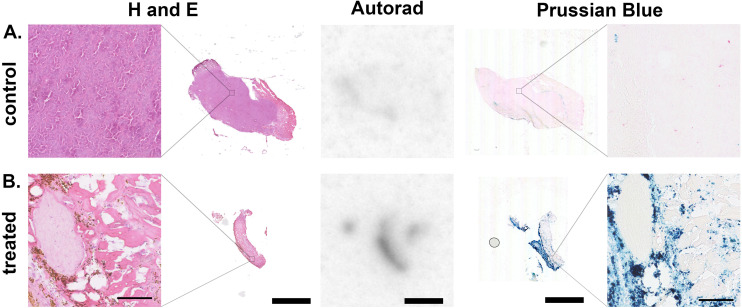
Representative contralateral (A) control (non-injected) and (B) ^111^In-RCL-01 injected tumours from the same animal, with serial sections showing co-localisation of ^111^In (autoradiography) with iron oxide nanoparticles (Prussian blue stain), together with a corresponding change in tissue morphology (H and E stain) and tumour size post-hyperthermia. Tissue was removed 5 days post hyperthermia therapy, and cryo-sectioned at 20 μm thickness. Thin scale bars = 100 μm, thick scale bars = 2.5 mm.

To confirm the co-location of radionuclide signal and RCL-01 iron oxide nanoparticles, control (uninjected, Fig. 4A) and injected (Fig. 4B) tumours were removed and processed for histology at 5 days post treatment. Comparison of autoradiography and Prussian blue stained sections showed a similar distribution of ^111^In and iron oxide particles in the samples, respectively, consistent with the maintained binding of the radiolabel to the nanoparticle (Fig. 4). To further validate ^111^In-based estimates of tissue iron content, this was independently measured using a ferrozine-based assay on samples of liver, spleen, and tumour tissue (Table S4†). This gave good agreement between the two measures with an *R*^2^ = 0.93, supporting ^111^In SPECT as a valid tool for non-invasive particle quantification. H and E staining showed tissue-level changes in the treated tumours compared to the control tumours, as well as a reduction in size, consistent with the larger scale changes observed using CT (Fig. 4A, B and Fig. S6†).

### Off-target accumulation is within safe limits

**Fig. 5:**
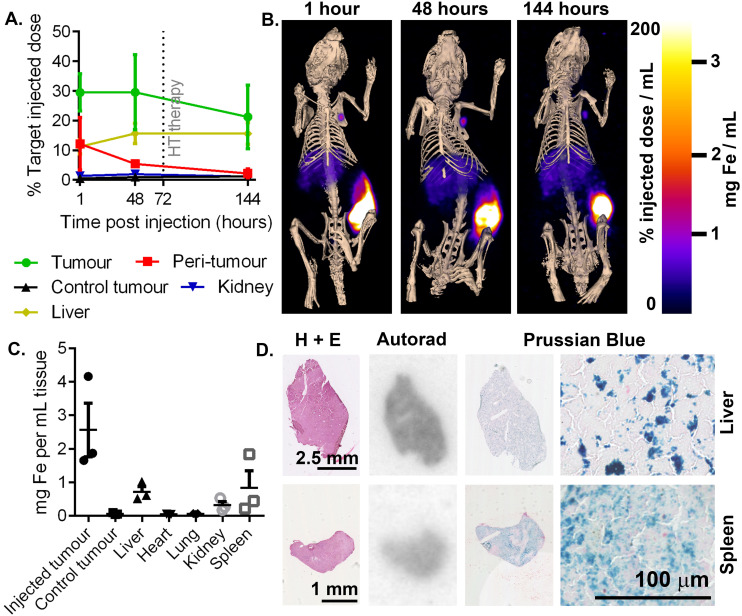
(A) Region-of-interest quantification of ^111^In-RCL-01 distribution based on SPECT-CT acquisitions from multiple time points post injection using ultrasound-guidance and syringe-pump controlled injection rate (*n* = 3, error bars show SD). (B) Maximum intensity projection SPECT images overlaid on 3D bone rendering, showing distribution of ^111^In-RCL-01 as in (A). The injected tumour, followed by the liver, show the highest uptake concentration at all time points. (C) *Ex vivo* estimates of iron concentration based on gamma-counter quantification of ^111^In-RCL-01 in multiple organs at dissection at 8 days post injection. (D) Confirmation of ^111^In-RCL-01 in liver and spleen, *via* autoradiography (^111^In), and Perl's Prussian blue stain (Fe) on 20 μm sections at 8 days post injection.

To assess the risk of off-target damage to the healthy tissues with high nanoparticle uptake, tissue concentrations of injected ^111^In-RCL-01 nanoparticles were quantified by SPECT imaging. The highest off-target uptakes were found to be in the liver (16% ± 6% SD) and kidneys (2% ± 1% SD) (Fig. 5A) at 48 hours post injection. In addition to the liver being particularly visible at each time point on SPECT-CT, uptake suggesting clearance to the axillary lymph node is also notable on the side of the injected tumour (Fig. 5B). *Ex vivo*^111^In measurements were also made at 8 days post-injection on dissected organs, which confirmed the liver and kidneys as two of the tissues with high uptake following the tumour, together with the spleen. These values were converted to iron content per gram of tissue for each organ (Fig. 5C), confirming that following the tumour, uptake was next highest in the liver and spleen. The presence of both the ^111^In and iron components of the labelled nanoparticles was confirmed with autoradiography and Prussian blue staining on serial tissue sections (Fig. 5D).

To predict clinical safety, we extrapolated iron accumulation values in patients based on scaled up organ sizes and dose (Table 1). The maximal predicted off-target heating rate under known clinical AMF conditions was *ca.* 4 mW g_tissue_^−1^ (in the spleen), which is well below the recommended limit of 20 mW g_tissue_^−1^ for nonspecific heating of tissues in the torso.^46^ Based on these values, significant off-target heating of these organs would not be expected, with the iron concentrations being well below those needed to give measurable heating in tumours under typical therapeutic AMF settings.

**Table 1:** Predicted upper limits of off-target iron accumulation from a clinical dose of RCL-01 and the corresponding worst-case specific absorption rates (SAR) in those organs. Organ concentration values estimated using *ex vivo* measurements of the percentage injected dose per organ at 8 days post injection of RCL-01 and scaled up to a clinical dose of 0.5 mL RCL-01 (at 75 mg_Fe_ mL^−1^) and reference organ weight ranges. Specific absorption rate (SAR) values estimated using AMF parameters currently being used, with RCL-01, in the NoCanTher clinical study in Barcelona, *viz*. an AMF of amplitude 4 kA m^−1^ and a frequency of 330 kHz

	Male				Female			
	Fe concentration (mg_Fe_ g_tissue_^−1^)		SAR (mW g_tissue_^−1^)		Fe concentration (mg_Fe_ g_tissue_^−1^)		SAR (mW g_tissue_^−1^)	
	Value	±SD	Value	±SD	Value	±SD	Value	±SD
Liver	0.006	0.003	0.2	0.1	0.007	0.003	0.2	0.1
Kidney	0.026	0.013	0.7	0.3	0.036	0.017	0.9	0.5
Spleen	0.125	0.074	3.3	1.9	0.163	0.096	4.3	2.5
Heart	0.004	0.002	0.1	0.1	0.005	0.003	0.1	0.1
Lung	0.001	0.001	<0.1	<0.1	0.001	0.001	<0.1	<0.1

## Methods

### Nanoparticle radiolabelling

Multicore magnetic nanoparticles (400 μL RCL-01, *ca.* 140 nm diameter particles comprising *ca.* 10 nm diameter iron oxide nanoparticles encapsulated in a dextran matrix, suspended in water for injection at concentration 75 mg_Fe_ mL^−1^; Resonant Circuits Limited) were mixed with 125 μL ^111^InCl_3_ (*ca.* 100 MBq) and 10 μL 1 M sodium hydroxide and heated for 2 hours at 90 °C. Labelling was confirmed using thin layer chromatography (TLC) with 500 μL DTPA solution (50 mM, pH 7.4) as the mobile phase and aluminium foil-backed silica gel matrix strips (1 cm × 10 cm × 200 μm; Sigma Aldrich) as the stationary phase. ^111^InCl_3_, and equivalent unheated particle/^111^InCl_3_ solutions were used as negative controls. TLC strips were cut at *R*_f_ 0.1 and activity from each section quantified using a gamma counter (Wizard2, PerkinElmer). Radiochemical purity was calculated as the percentage of the total activity from both sections of the strip that was below *R*_f_ 0.1. The same method of labelling was also followed for ^89^Zr, using a stock solution of ^89^Zr-oxalate (PerkinElmer).

To measure stability, particles labelled as above and diluted to a concentration of 20 MBq per mL in human serum (Standard Pooled Human Serum, Cambridge Bioscience), with 500 U mL^−1^ penicillin–streptomycin (Gibco), and incubated at 37 °C. Aliquots were taken at 1, 2, 3, 4, and 7 days, incubated for 120 minutes at 37 °C with 10 mM DTPA, and analysed by TLC as above.

### SQUID magnetometry and *in vitro* heating assay

RCL-01 particles (5 mg Fe) were reacted at 90 °C for 90 minutes in a volume of 500 μL containing either 2 mM ZrCl_4_, InCl_3_, or without metal additives, adjusted to pH 8 using NaOH. Following the reaction period, 100 μL HEPES buffer (pH 7, 200 mM) was added to neutralize the solution. Particles were used at a final concentration of 5 mg_Fe_ mL^−1^ for *in vitro* magnetic heating measurements.

Magnetisation data was recorded on 5 mg mass air-dried samples using a Quantum Design MPMS Superconducting Quantum Interference Device (SQUID) VSM Magnetometer (San Diego, USA) at 300 K over a field range of ±70 kA m^−1^.

Hyperthermia experiments were undertaken using a Materials Characterisation MACH (Magnetic Alternating Current Hyperthermia) system designed and built by Resonant Circuits Limited. This system comprised a six-turn water-cooled copper-pipe solenoid which was driven by a high *Q*-factor resonant controller and produced near-homogeneous fields (within the solenoid) of amplitude up to 8.5 kA m^−1^ and a fixed frequency of 990 kHz. Measurements were performed on liquid suspensions, following best practice guidelines.^30^ Sample temperatures were monitored using an optical fibre mounted fluoroptic temperature probe (Luxtron FOT Lab Kit, Lumasense, California, USA).

### Cell culture

A-375 human malignant melanoma cells (obtained from Cancer Research UK) were grown in DMEM (Gibco) supplemented with 10% FBS (Gibco), at 37 °C in 5% CO_2_. When 80% confluent, cells were washed with PBS solution (Gibco), detached *via* incubation with a 0.05% trypsin EDTA solution (Gibco), and re-seeded at 1 in 10 concentrations in fresh DMEM medium.

### Tumour implantation

Female SCID mice (Charles River) were used for all experiments at 3 months old. Tumours were grown on each rear flank following subcutaneous implantation of 1 × 10^6^ A-375 cells per flank. Tumour growth was monitored using palpation, caliper measurements, and X-ray CT as described below. All animal studies were approved by the University College London Biological Services Ethical Review Committee and licensed under the UK Home Office regulations and the Guidance for the Operation of Animals (Scientific Procedures) Act 1986 (Home Office, London, United Kingdom). All animal methods were performed in accordance to institutional ethical guidelines and regulations.

### CT imaging of tumour volume

Mice were anaesthetized with 1.5 to 2.5% isoflurane in oxygen mixture, and a small animal physiological monitoring system (SA Instruments, Stony Brook, NY) was used to monitor respiration rate and temperature, which was kept at 37 °C using a heated bed. CT images were acquired with tube voltage of 55 kV (peak), 500 ms exposure time, binning of 1 : 4, and 180 projections, using a NANOScan (Mediso) CT imaging device one day before nanoparticle injection. Volumes of interest (VOIs) were drawn over the left and right tumour using VivoQuant software, with the soft-tissue contrast indicating the tumour boundaries.

### Caliper measurements

Mice were anaesthetized and long diameter (length) and short diameter (width) measurements were made with digital calipers from when tumours first became palpable. Tumour volume was estimated as π/6 × (*L* × *W*^2^), where length is the long axis measurement.

### Nanoparticle injection

For all injections, target doses were calculated in μL as one third of the total current volume of the tumour, which was measured using CT the day prior to injection. Appropriate volumes of ^111^In-RCL-01 particles were loaded into 1 mL syringes, fitted with either 27 gauge needles alone (for manual injections), or, for ultrasound-guided injections, 27 gauge needles at the end of catheters made from fine bore polythene tubing (0.38 mm ID, 1.09 mm OD, Portex, Ref. 800/100/120). For syringe-pump controlled injections a flow rate of 5 μL per minute was achieved using a programmable syringe pump (Harvard Apparatus, PHD 4400 Hpsi). Ultrasound-guided injections were performed using a FujiFilm Visualsonics Vevo 2100 with MS550D ultrasound transducer and injection mount.

### SPECT-CT imaging

Mice were anaesthetised with 1.5 to 2.5% isoflurane in oxygen mixture, and a small animal physiological monitoring system (SA Instruments, Stony Brook, NY) was used to monitor respiration rate and temperature, which was kept at 37 °C using a heated bed. Mice were imaged with intervals between 0 to 30 days post nanoparticle injection, using the PinSPECT (In 111) acquisition setting. All images were acquired with same range of CT scan and 45 seconds termination condition per frame, using NanoSPECT/CT (Mediso). Volumes of interest (VOIs) were drawn over the left and right tumour, as well as liver and kidney, using VivoQuant software using the CT soft-tissue contrast as a guide. The amount of radioactivity in the tumour and around it, as well as uptake in the liver and kidneys, was derived from the VOIs.

### Hyperthermia treatment

Mice were anaesthetized and placed in the centre of a water-cooled whole-body coil of a Preclinical MACH (Magnetic Alternating Current Hyperthermia) system designed and built by Resonant Circuits Limited. This system comprised a six-turn split pair assembly (two co-axial sets of three turns, separated on axis by a gap of 15 mm, designed for ease of access and data collection in animal experiments) of water-cooled copper pipe which was driven by a high *Q*-factor resonant controller and produced near-homogeneous fields (within the gap) of amplitude up to 5.47 kA m^−1^ and a fixed frequency of 940 kHz. For the *in vivo* animal experiments, the system was run for 10 minutes at an amplitude of 4.21 kA m^−1^, followed by 20 minutes at amplitude of 5.47 kA m^−1^, in both cases at a frequency of 940 kHz. Body temperature was monitored using a rectal probe and maintained as close to 37 °C as possible using a heat lamp. An infrared thermal imaging camera (InfraTec VarioCAM® HR Research 780) with IRBIS 3 software (InfraTec, Dresden, Germany) was used to monitor the surface temperature of the tumours, to ensure that this did not rise above *ca.* 50 °C.

### Histology and autoradiography

Excised tissues were embedded in OCT media (Tissue-Tek) and flash-frozen in a bath of isopentane cooled on dry ice. Samples were then mounted and cut using a cryomicrotome (Leica CM 3050) at a thickness of 20 μm. These were then fixed on glass slides (Superfrost Plus, Thermo Scientific) in a 4% buffered solution of formalin for 10 minutes, before being washed three times in phosphate buffered saline (PBS). Fixed slides were then used for autoradiography, followed by either Prussian blue staining or H and E staining.

For autoradiography, 20 μm thick tissue sections were fixed into a cassette and exposed to a phosphor storage plate for 24 hours (GE Healthcare). Plates were removed and scanned using a Typhoon imaging device (Amersham) at 50 μm resolution, and maximum sensitivity setting. Files were exported and analysed using ImageJ software (NIH).

A Prussian blue staining protocol was used to detect the distribution of RCL-01 particles in tissue. Sections prepared as above were put into 2% potassium ferrocyanide solution mixed 1 to 1 with 2% hydrochloric acid solution for 20 minutes. Slides are then rinsed with water before counter staining with nuclear fast red for 30 seconds, before dehydration through a range of ethanol concentrations and mounting with DPX mounting media. H and E staining was done using an automated staining device (Tissue-Tek, DRS), also followed by DPX mounting. Images of Prussian blue and H and E stained slides were taken using a Nanozoomer slide imaging device (Hamamatsu, China) and analysed using NDP View 2 software (Hamamatsu).

### *Ex vivo* iron assay

Selected tissues removed at 8 days post injection (15–50 mg per sample wet weight) were ground to a powder consistency in liquid nitrogen using a liquid nitrogen-cooled mortar and pestle. Iron concentration was estimated using a ferrozine-based colorimetric assay (MAK025, Sigma Aldrich) according to the manufacturer's instructions, with sample absorbance measured using a spectrophotometer (Varioskan Lux, Thermo Fisher). Experimental samples were measured at multiple dilution factors to enable fitting of readouts to the linear range of the standard curve.

## Discussion

Many factors contribute to failures in cancer treatment, including tumour heterogeneity, drug resistance, invasion of healthy tissue, and the dose-limiting side effects of drugs and radiation.^47–49^ Magnetic hyperthermia has the potential, in at least some scenarios, to address these challenges,^11,50^ whilst simultaneously boosting responses to, and lowering necessary doses of immunotherapy, chemotherapy, or radiotherapy when used in combination.^40,51^ However, for magnetic hyperthermia to reach routine clinical use, accurate and reliable delivery technologies must be developed, and their efficacy quantified.

To this end, the results presented here suggest that ultrasound-guided delivery, together with syringe-pump controlled injection rate, is a feasible and effective method to improve the accuracy and efficacy of iron oxide nanoparticle delivery, when compared to manual injection. By enabling direct visualization of the tumour and surrounding tissue in real time during needle placement, it provides a route towards a more personalised delivery protocol, factoring in differences in tumour shape and size, respiratory-related movement, and operator error. For wider translation of this technique, the range of clinically established ultrasound devices covers several potential tumour locations beyond the more superficially located tumours studied here. For example, needle insertion, or focal delivery under ultrasound guidance is clinically established using transrectal ultrasound (TRUS) for prostate tumours,^45,52^ while the pancreas can be targeted by endoscopic ultrasound devices.^53^

Though we did improve delivery several-fold *versus* manual injection, one limitation of the present study was the modest overall percentage of the target dose (<50%) that was delivered intratumorally, even following optimisation. A contributing factor to this was the frequently observed backflow of injection material over the outside of the needle tip (also known as reflux), and out of the site of insertion on the dermis. This problem of reflux has been well-described and modelled, with multi-hole or “convection-enhanced” needle tips, as well as injection rate, being among the solutions shown to enhance delivery in high pressure tumours.^54–57^ Interstitial tumour pressure and stiffness has previously been shown to vary over orders of magnitude, and to affect drug delivery.^54,58^ Various strategies to reduce interstitial tumour pressure have therefore been shown to improve drug delivery,^59–61^ suiting this approach for future combination with guided-delivery and the convention-enhanced needle tips described above. To quantify the effectiveness of such a combined approach we hope to make further use of the heat-induced radio-labelling method and improve upon the methods developed here in more representative orthotopic tumour models. With tumour stiffening or fibrosis having been noted as a side effect of both hyperthermia treatment^62^ and brachytherapy,^45^ application of such advanced delivery strategies for patients undergoing multiple therapies might become yet more important.

One previous study on the delivery of nanoparticles for hyperthermia therapy showed better treatment results following 5 μL min^−1^ injection rates *versus* 10 or 20 μL min^−1^,^63^ though delivery was not quantified. Nevertheless, our decision to use of this single, reportedly optimal injection rate (5 μL min^−1^) for the syringe-pump controlled condition might be considered a further limitation of our study. This is because, although one previous clinical report described the necessity of using slow injections when tumour tissue was stiff or fibrotic,^45^ optimal rates are likely to vary between tumour types and between preclinical models and human tumours, depending both on the physical characteristics of the tumour tissue, and needle type.

To avoid damage to healthy tissue during this study, we used AMF settings intended to keep surrounding healthy tissue below 50 °C during treatment, which we confirmed using real-time infra-red measurements. This trade-off between tumour heating, or ablation, and preservation of healthy tissue has been one of the constant challenges facing hyperthermia-based therapies. More sophisticated AMF application routines including pulsed field application have been shown to achieve a better balance between heating the tumour and healthy tissue,^64,65^ as well as increasing tumour response for a given thermal dose.^29^ Though here we only used a basic hyperthermia protocol of continuous AMF application, use of a pulsed protocol would be another method to seek to go beyond the mean decrease in tumour volume of 58% achieved in this study, without further optimisation of delivery.

Though we only used a single nanoparticle type in this study, tumour-localized delivery is key to the therapeutic mechanism of a range of nanoparticle-based therapies, with our results clearly suggesting the benefit that ultrasound guided delivery could have beyond the field of hyperthermia. Aside from the many nanoparticle-based drug platforms for enhancing small molecule delivery, nanoparticles are also being developed for a range of other more advanced therapeutic strategies, including as radiosensitisers,^66^ as radiotherapeutic agents such as in brachytherapy,^67,68^ and with photothermal and photodynamic therapies.^69^ In the majority of these applications, the degree of treatment response, and limitation of side effects, could be improved by optimizing injection strategies as demonstrated here. We have previously shown the compatibility of this labelling approach with a range of commercially available iron oxide nanoparticles with varying size, material, and coating, supporting the wider use of this technology.^28^ Beyond nanoparticle-based therapies, we have also recently demonstrated imaging-based approaches to measuring the delivery of hydrogels, microspheres, and/or stem cell to the kidneys and heart, also using ultrasound guidance to ensure correct needle placement.^70–72^

The value of imaging delivery has previously been highlighted in clinical trials, with CT used to follow the distribution of nanoparticle-based hyperthermia agents in both prostate tumours and glioblastoma.^45,73,74^ In these studies, as we did here, the particle dose and distribution allowed pre-treatment planning to reduce the risk of damaging surrounding healthy tissue. However, the limited sensitivity of CT technique has also been highlighted,^24^ preventing accurate quantification in areas of low accumulation, and prompting the development of more sensitive, radionuclide-based approaches such as that demonstrated here. While useful for short term-tracking of nanoparticles, the ∼3 day half-life of ^111^In and ^89^Zr does however limit the period of tracking to around 2 weeks, providing useful data only for the period between the initial injection and application of heating. Longer term tracking of particle fate, where clearance has occurred and lower concentrations remain in the tumour, still leaves a role for other imaging modalities such as MRI, with its high sensitivity to magnetic particles,^15^ and lack of half-life related signal decay. While ^111^In SPECT was favoured here due to its higher preclinical resolution *vs.*^89^Zr PET, the converse however is true in the clinic. Here, PET not only provides better resolution than SPECT, but also orders of magnitude higher sensitivity with the newer whole-body imaging devices, with tracking of ^89^Zr-labelled antibodies shown up to 30 days post-injection in patients.^75^ Together with ability to use orders of magnitude lower doses of radiation, ^89^Zr labelling of RCL-01 (Fig. S3 and S4†) for PET would therefore be the preferred route for translation.

Though not currently available in the clinic, Magnetic Particle Imaging (MPI) could arguably provide additional advantages to SPECT or PET, while avoiding the need for radiolabelling. Relying directly on the presence of superparamagnetic iron oxide particles for contrast, it combines high spatial resolution (<1 mm) with sufficient dynamic range for imaging MFH-relevant iron concentrations.^25^ With much of the hardware needed for generating therapeutic AMF fields already present in standard MPI equipment, together with the use of similar RF frequencies, combination of MFH and MPI functionality within the same device has been shown to have several benefits aside from convenience.^76–79^ Firstly there is greater AMF field spatial control with MPI devices *vs.* traditional dedicated MFH coils, reducing off-target heating of distant organs such as liver and spleen.^80^ Secondly, the thermal sensitivity of MPI signal response allows real-time monitoring of tissue heating to allow better control and prediction of thermal dose during therapy.^80,81^ Further applications such as real-time monitoring of intravascular interventions are also emerging, offering a route to image-guided drug delivery.^82^ However MPI is not without its limitations, among which is the complex relationship between signal and particle content within a given voxel *in vivo*. Intra-particle, and inter-particle effects (including aggregation), as well as degradation can vary over time and between and within tissues in the body, influencing MPI signal in ways that are difficult to predict – sometimes raising it, sometimes lowering it compared to pristine, freely dispersed particles at the same concentration.^83,84^ These effects also differ between particles of different cores and coatings, with further research needing to be done to ensure the reliability of particle quantification *in vivo*.

Despite the measurable accumulation of nanoparticles in the liver and spleen, our measurements and projections of human doses gave confidence that this was below the level needed to produce off-target heating, therefore informing on the safety as well as the efficacy of this therapeutic approach. With off-target delivery, and consequent side-effects being one of the main limiting factors in the success of many established cancer therapeutics, this is an important finding supporting the translation of RCL-01.

As the prognosis of patients with late stage, treatment resistant melanomas remains poor,^32,33^ the development, optimisation, and validation of novel treatments such as this offers an important route toward improved patient outcomes. In this regard the 58% reduction in tumour size at 3 days post hyperthermia achieved here was highly promising. Owing to our choice of a human melanoma line however, these results were obtained in immune compromised animals, preventing combination in this study with an immunotherapy approach. Previous reports have shown synergistic effects of combining hyperthermia therapy with immunotherapy or radiotherapy,^35–39^ providing scope for future work to improve upon the results obtained here.

In summary, we have shown that ultrasound guidance, together with slow, controlled injection rate, can improve both the degree, and accuracy of nanoparticle delivery in a mouse model of melanoma. We have quantified this improvement using a recently developed radiolabelling technique and SPECT imaging, giving high resolution data on particle distribution across the body. This illustrates the value of this labelling and imaging approach in obtaining further optimisation of iron oxide nanoparticle delivery, whether for application in other disease areas, or using other advanced delivery strategies such as engineered needle tips. Hyperthermia treatment was highly effective at reducing tumour size following the optimized injections, while off-target delivery to the liver and spleen was shown to be within safe limits. Together, this supports the possible role for the RCL-01 magnetic heating agent in addressing additional unmet needs in cancer therapies, beyond the ongoing clinical trial in pancreatic cancer patients.

## Conflicts of interest

Some authors are connected with Resonant Circuits Limited (RCL), a start-up company active in the field of magnetic thermotherapy, *viz.*: P. S. and S. R. H. are both employees of RCL, and Q. A. P. acts, in an independent capacity, as a consultant to RCL.

## Supplementary Material

NR-016-D4NR00240G-s001
