# Supplementary material for: Improved tumour delivery of iron oxide nanoparticles for magnetic hyperthermia therapy of melanoma via ultrasound guidance and 111In SPECT quantification
Source: Nanoscale. 2024 Jul 17;16(42):19715–29. doi: 10.1039/d4nr00240g (PMC11488578; doi:10.1039/d4nr00240g)
Supplement: NR-016-D4NR00240G-s001 [file NR-016-D4NR00240G-s001.pdf]

Electronic Supplementary Material (ESI) for Nanoscale.
This journal is © The Royal Society of Chemistry 2024

Supplementary Information

Improved Tumour Delivery of Iron Oxide Nanoparticles for Magnetic Hyperthermia Therapy of Melanoma via Ultrasound Guidance and ^{111}In SPECT Quantification

P. Stephen Patrick [a*], Daniel J. Stuckey [a], Huachen Zhu [a], Tammy L. Kalber [a], Haadi Iftikhar [b], Paul Southern [b,c], Joseph C. Bear [d], Mark F. Lythgoe, Simon R. Hattersley [c], Quentin A. Pankhurst [b,c]

a. Centre for Advanced Biomedical Imaging (CABI), Department of Medicine, University College London, London WC1E 6DD, UK

b. Healthcare Biomagnetics Laboratory, University College London, 21 Albemarle Street, London, W1S 4BS, UK

c. Resonant Circuits Limited, 21 Albemarle Street, London, W1S 4BS, UK

d. School of Life Science, Pharmacy & Chemistry, Kingston University, Penrhyn Road, Kingston upon Thames, KT1 2EE, UK

* Corresponding author: peter.patrick@ucl.ac.uk

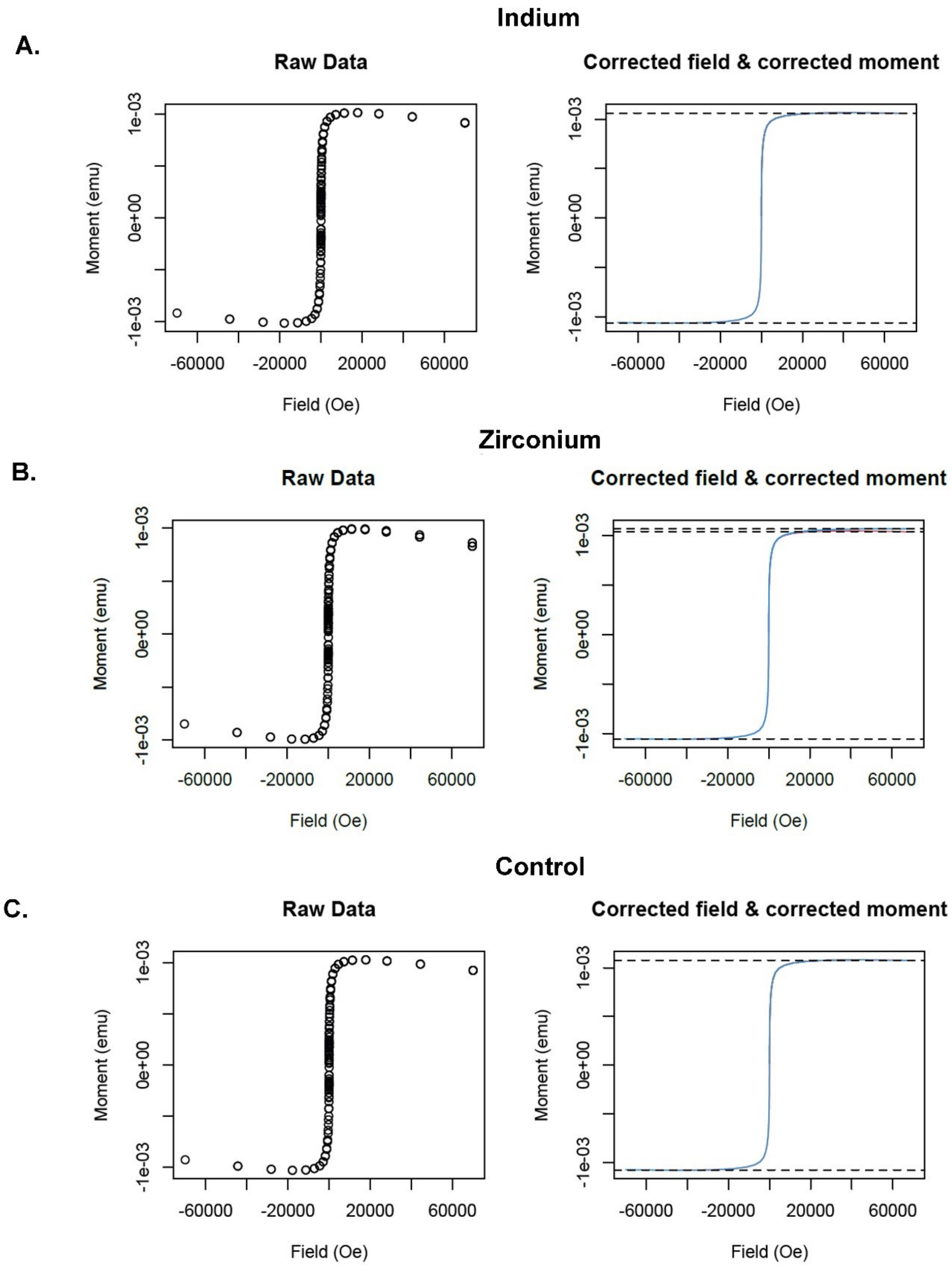

Figure S1. Magnetic properties are retained by RCL-01 multicore iron oxide in dextran magnetic nanoparticles post heat-induced labelling with **A.** indium and **B.** zirconium, as compared to **C.** unlabelled nanoparticles. Data shown are magnetization curves as measured by SQUID magnetometry from a 5.0 mg mass of air-dried particles: both the raw data, as measured, and the corrected data, after accounting for diamagnetic background signals and minor flux pinning effects. As can be seen from the data, the same saturation magnetic moment – 0.0011 emu – was measured for each sample.

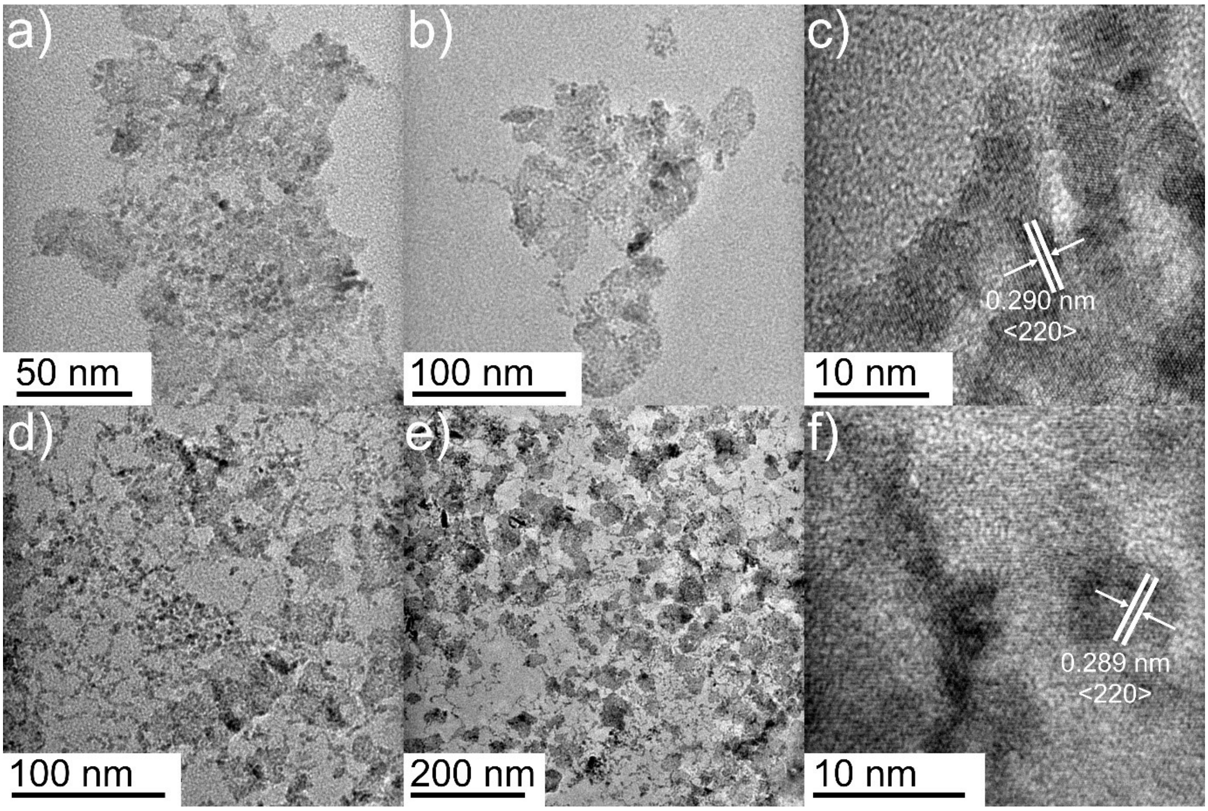

Figure S2. Transmission Electron Micrographs showing RCL-01 a) Indium doped, b) Zr-doped, c) Zr-doped high resolution, d) Stock, e) stock, f) stock high resolution.

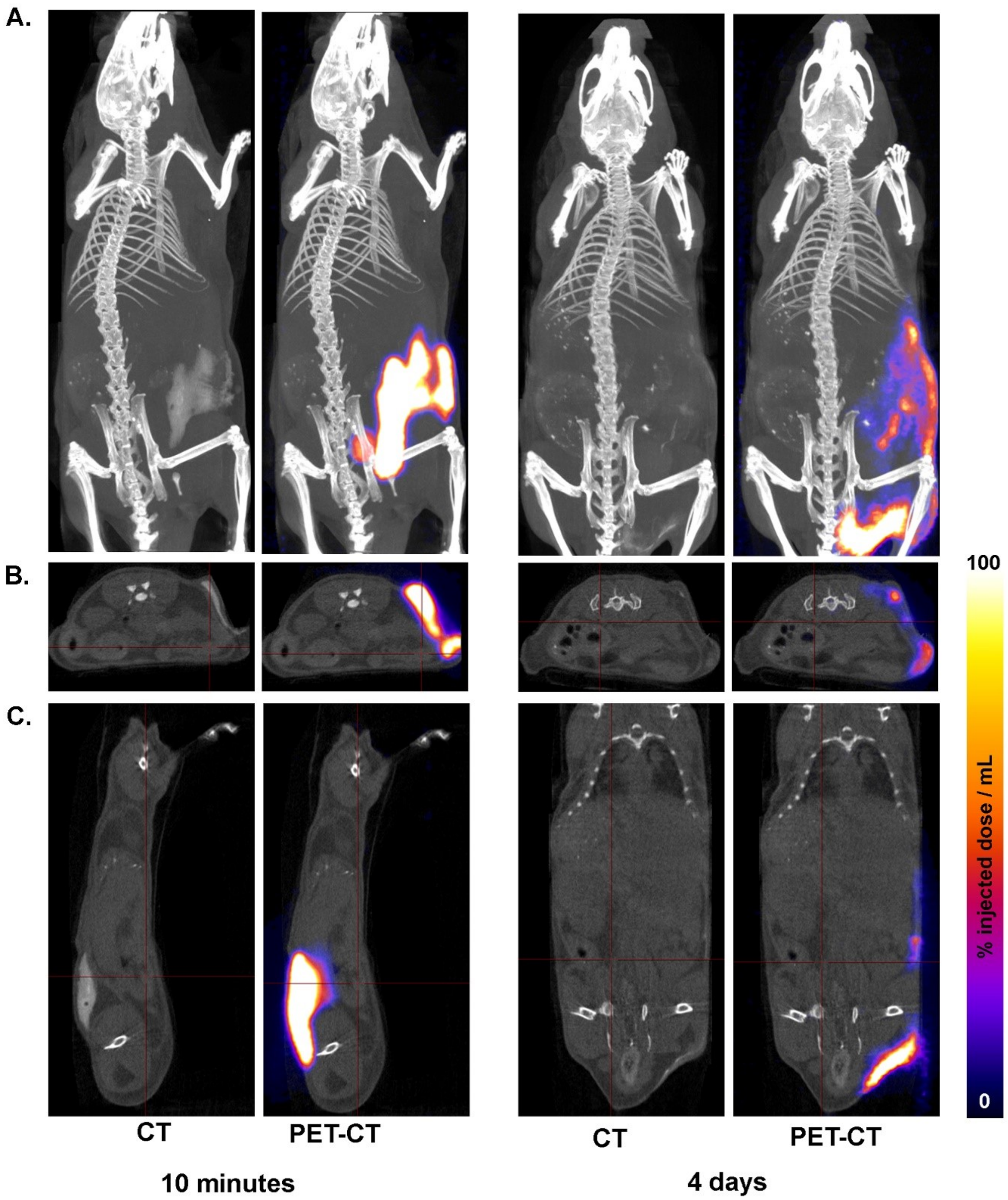

Figure S3. PET-CT images shown as **A.** maximum intensity projections, **B.** axial, and **C.** sagittal sections, following subcutaneous injection of 5 mg of ^{89}Zr-labelled RCL-01 magnetic nanoparticles. Images recorded at 10 minutes and 4 days post injection. Co-localisation of ^{89}Zr and CT signal, as well as lack of bone signal, demonstrates radiolabelling stability *in vivo*.

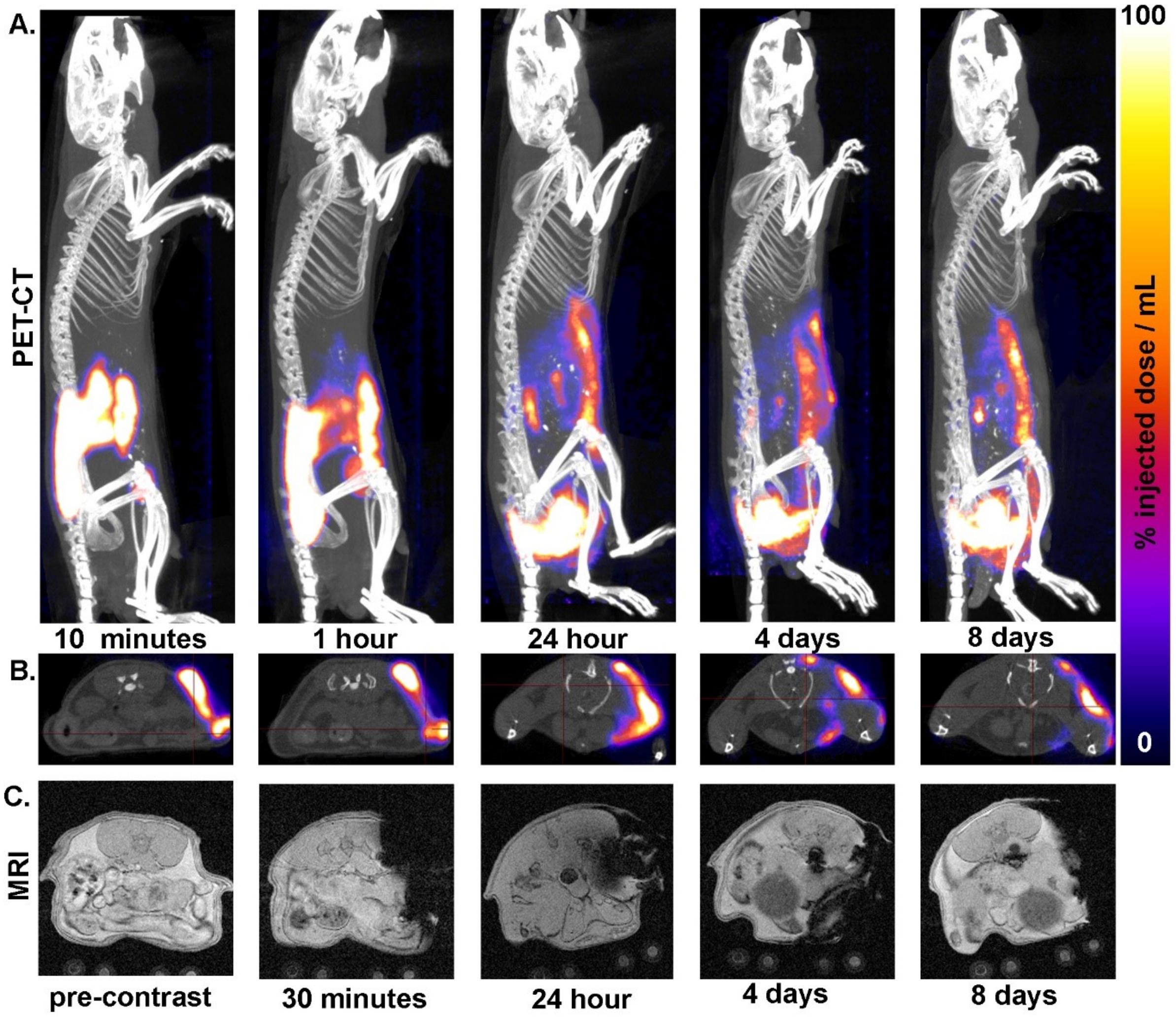

Figure S4. PET-CT images shown as **A.** maximum intensity projections, and **B.** axial sections, as well as **C.** axial MRI sections (1.5 mm, T_1-FLASH, TE 2 ms, TR 500 ms, Bruker 1T Icon) following subcutaneous injection of 5 mg of ^{89}Zr-labelled RCL-01 magnetic nanoparticles. Images recorded before injection, and up to 8 days post injection.

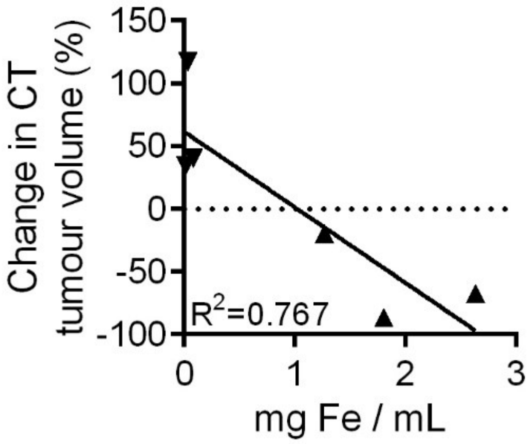

Figure S5. Correlation between the amount of injected iron (associated with the RCL-01 magnetic nanoparticles) per unit tumour volume, measured in mg_{Fe}/mL_{tissue}, as estimated by SPECT CT analysis 1 day pre HT, and the percentage change in tumour volume at 3 days post HT therapy.

A.

B.

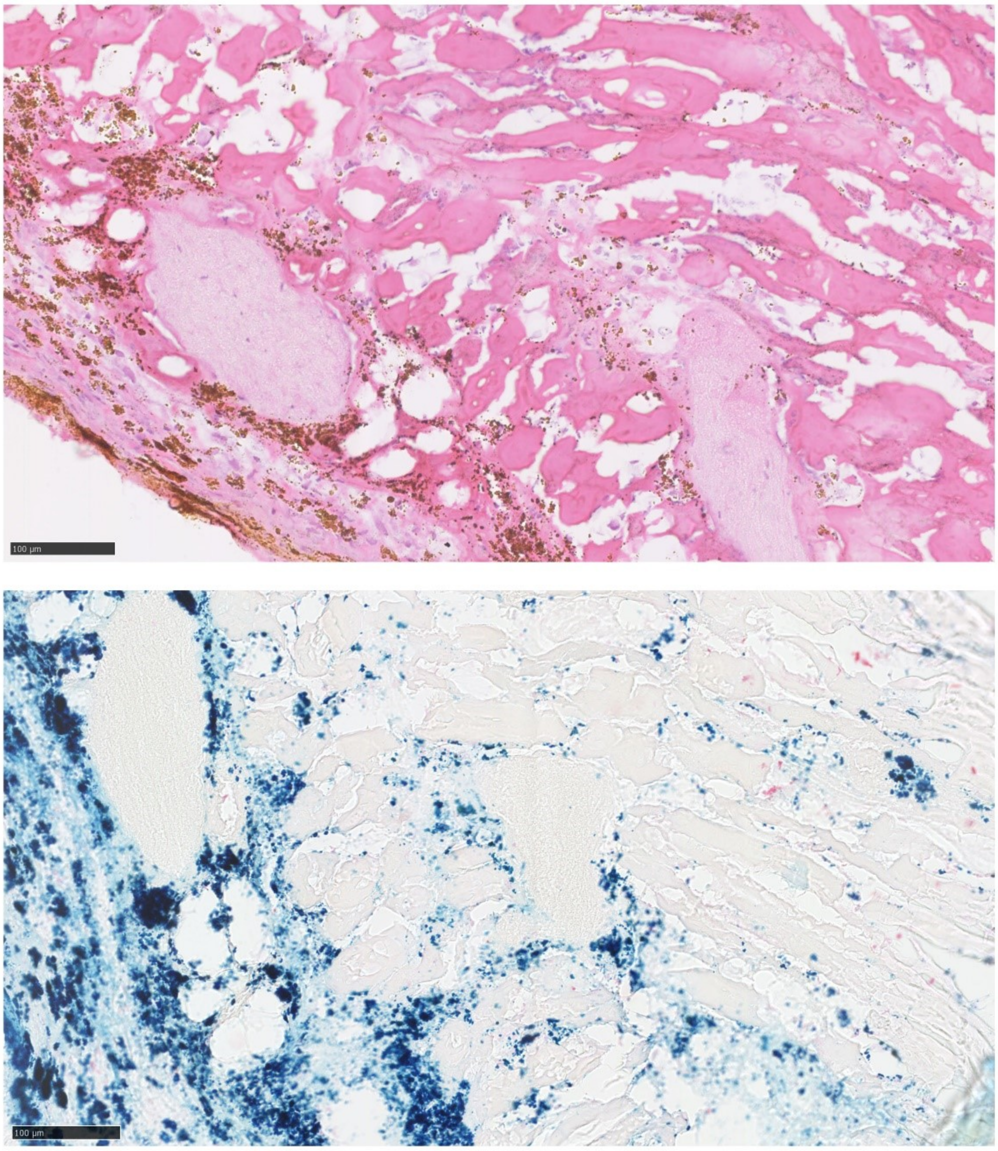

Figure S6. Areas of tissue damage and nanoparticle deposition respectively shown in corresponding **A.** H and E (haemotoxylin and eosin), and **B.** Perl's Prussian Blue (for iron) stained sections of tumour tissue removed 5 days post hyperthermia therapy, and cryo-sectioned at 20 µm thickness.

Condition	^{111}In % RCY (+/- SD)	^{89}Zr % RCY (+/- SD)
RCL-01 (heated at 90°C)	96.3 (2.5)	98.1 (1.7)
RCL-01 (unheated)	3.2 (0.7)	9.4 (5.2)
Radiometal Stock (control)	2.9 (0.4)	8.8 (6.1)

Table S1. Thin layer chromatography (TLC) analysis showing heat-induced radiolabelling of RCL-01 iron oxide-based nanoparticles with ^{111}In and ^{89}Zr (n=3 per condition). Percent radiochemical yield was measured as retention at the origin of aluminium foil-backed silica gel matrix strips, with DTPA solution (50 mM, pH 7.4) as the mobile phase. Control conditions (unheated with particles, or stock radiometal solution only) showed minimal retention.

Days post tumour implantation	19	20	20	22	26
				(1 day pre HT)	(3 day post HT)
Mouse	Tumour volume mm^3	Target injection volume mm^3	Estimated injected volume mm^3	Tumour volume mm^3	Tumour volume mm^3
M1	465	155	33	571	78
M2	135	45	19	381	124
M3	243	81	20	455	364

Table S2. Tumour volumes as measured by X-ray CT and region of interest analysis pre and post injection and hyperthermia therapy. Target injection volumes were based on one third of the tumour volume measured on the day before injection, with estimated actual injection volume estimated by SPECT analysis of activity in the tumour at 1 hour post injection. All nanoparticles injected at 41 mg_{Fe}/mL concentration.

Mouse	Liver	Heart	Kidney	Spleen
M1	1.020412	0.050832	0.526845	1.836082
M2	0.612244	0.044560	0.257807	0.441702
M3	0.519827	0.038248	0.173600	0.217550

Table S3. Estimated iron content (mg_{Fe}/g tissue) based on ex vivo ^{111}In gamma counter measurements of excised tissue taken at 8 days post injection.

	Ferrozine assay	Gamma counter
	mgFe/g	mgFe/g
Tumour (RCL01 injected) M2	3.474	4.158
Tumour (RCL01 injected) M3	1.19	1.655
Tumour (contralateral uninjected) M2	0.098	0.053
Tumour (contralateral uninjected 2) M3	0.103	0.041
Liver RCL01 M1	1.326	1.02
Liver RCL01 M2	0.715	0.612
Spleen RCL01 M3	0.972	0.217
Control liver (uninjected animal)	0.163	N/A
Control spleen (uninjected animal)	0.698	N/A
Control tumour (uninjected animal)	0.084	N/A

Table S4. Iron content (mg_{Fe}/g tissue) measured using the Ferrozine assay on selected excised tissue taken at 8 days post-injection of ^{111}In-RCL-01, with gamma-counter based

measurements of ^{111}In-RCL01 from the same tissue for comparison. Measurements correlated well with an R^2=0.927.

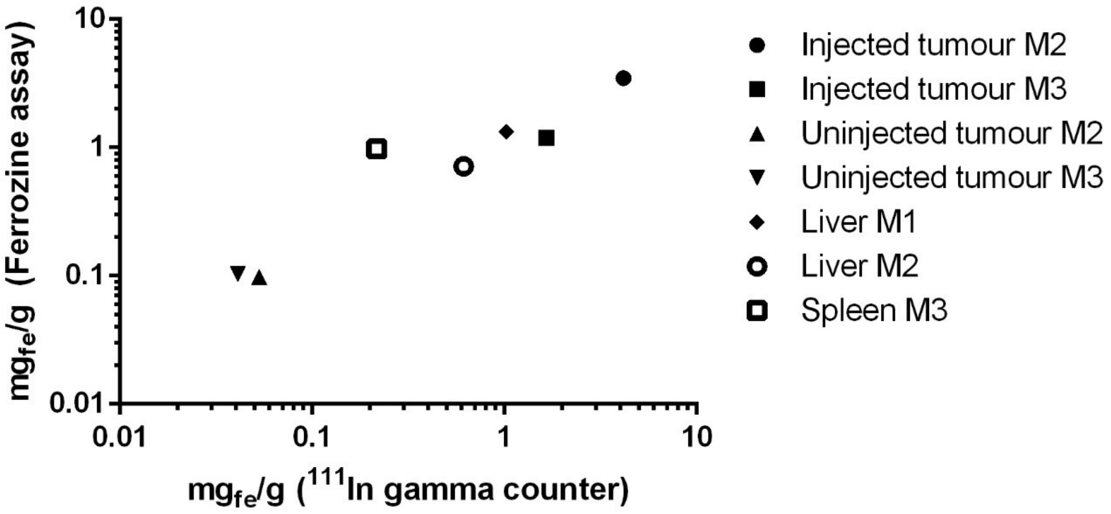

Figure S7. Comparison of mgFe/g tissue estimates from *ex vivo* ferrozine and ^{111}In gamma counter measurements on selected organs, showing good correlation **(**R^2=0.927). Note that the higher spleen value obtained by the ferrozine assay vs the ^{111}In estimate can be explained by the higher background iron content of this organ found in control (uninjected) tissue (Table S4).

Supplementary Methods

PET-CT

Mice were imaged at the stated time points after subcutaneous injection 5mg ^{89}Zr-RCL-01 (~10 MBq per mouse) using a PET-CT (Mediso NanoScan) interfaced to InterView Fusion software. CT was acquired at 50 kVp with 300-ms exposure and reconstructed in 0.13-mm isotropic voxels. PET data was reconstructed in 5:1 mode using the Tera-Tomo algorithm in 0.4-mm isotropic voxels and analysed using VivoQuant software (InViCro).

MRI

Magnetic Resonance Images were obtained using a 1T Brüker ICON desktop MRI system (Brüker BioSciences Corporation, Ettlingen, Germany), interfaced to an operating console running Paravision 5 software (Bruker). A 30-mm mouse body solenoid RF coil (Bruker) was operated on transmit/receive mode. A rectal thermometer and respiratory pad provided physiological monitoring (SA Instruments, New York, USA), with temperature maintained via a water-heated bed. A multi-slice T1-FLASH sequence was used to acquire 24 1.5mm serctions covering the area of injection (TE 2ms, TR 500ms).

TEM

Transmission electron microscope (TEM) images were obtained using a high resolution TEM Jeol 2100 with a LaB_6 source operating at an acceleration voltage of 200 kV. Images were recorded on a Gatan Orius Charge-coupled device (CCD). Samples were prepared by dipping a copper 400 mesh TEM holey carbon film grid (Agar Scientific Ltd.) held by tweezers into aqueous suspensions of the nanoparticles, before drying on filter paper.
